# Uniaxial Hydroxyapatite Growth on a Self-Assembled Protein Scaffold

**DOI:** 10.3390/ijms222212343

**Published:** 2021-11-15

**Authors:** Alexander L. Danesi, Dimitra Athanasiadou, Ahmad Mansouri, Alina Phen, Mehrnoosh Neshatian, James Holcroft, Johan Bonde, Bernhard Ganss, Karina M. M. Carneiro

**Affiliations:** 1 Faculty of Dentistry, University of Toronto, Toronto, ON M5G 1G6, Canada; a.danesi@utoronto.ca (A.L.D.); dimitra.athanasiadou@utoronto.ca (D.A.); ahmad.mansouri@mail.utoronto.ca (A.M.); alina.phen@mail.utoronto.ca (A.P.); mehrnoosh.neshatina@mail.utoronto.ca (M.N.); james.holcroft@utoronto.ca (J.H.); b.ganss@dentistry.utoronto.ca (B.G.); 2 Division of Pure and Applied Biochemistry, Center of Applied Life Sciences, Lund University, 223 62 Lund, Sweden; johan.svensson_bonde@tbiokem.lth.se; 3 Institute of Biomedical Engineering, University of Toronto, Toronto, ON M5S 3G9, Canada

**Keywords:** biomineralization, biomimetics, bio-inspired materials, amelogenin, amelotin hydroxyapatite, enamel

## Abstract

Biomineralization is a crucial process whereby organisms produce mineralized tissues such as teeth for mastication, bones for support, and shells for protection. Mineralized tissues are composed of hierarchically organized hydroxyapatite crystals, with a limited capacity to regenerate when demineralized or damaged past a critical size. Thus, the development of protein-based materials that act as artificial scaffolds to guide hydroxyapatite growth is an attractive goal both for the design of ordered nanomaterials and for tissue regeneration. In particular, amelogenin, which is the main protein that scaffolds the hierarchical organization of hydroxyapatite crystals in enamel, amelogenin recombinamers, and amelogenin-derived peptide scaffolds have all been investigated for in vitro mineral growth. Here, we describe uniaxial hydroxyapatite growth on a nanoengineered amelogenin scaffold in combination with amelotin, a mineral promoting protein present during enamel formation. This bio-inspired approach for hydroxyapatite growth may inform the molecular mechanism of hydroxyapatite formation in vitro as well as possible mechanisms at play during mineralized tissue formation.

## 1. Introduction

The formation of mineralized tissues is an intrinsically hierarchical process, where cells first deposit an extracellular matrix (ECM) that must achieve the appropriate nanoscale order to guide and promote mineral growth. Most mineralized tissues comprise hydroxyapatite (HAP) mineral of varying morphological characteristics and with a concentration ranging from 70% to 96% depending on the tissue [1]. In particular, tooth enamel is the hardest tissue in humans, composed of >95% HAP [2]. During the process of enamel formation, ECM proteins, predominantly amelogenins, in combination with mineral promoting proteins guide the formation of highly ordered, complex crystal structures before being degraded by proteases and almost completely removed from the tissue [3]. In vitro, recombinant protein, recombinamer, and peptide scaffolds systems based on amelogenin have also demonstrated mineral scaffolding capability [4,5,6,7,8,9,10]. Additionally, amelotin (AMTN), an enamel protein, has been shown to be a mineral promoter both in vitro and in vivo [11,12,13,14]. The mineral formation rate depends on factors such as the supersaturation, temperature, and organic matrix. For example, organic matrices and especially proteins can increase the nucleation rate and subsequent crystal growth [15]. Serving a role distinct from that of amelogenin during tissue formation, amelotin may enhance the in vitro mineral growth capabilities of amelogenin-based scaffold materials.

Previously, we engineered a recombinamer protein containing a well-known mineral promoting peptide sequence found within both amelotin and the Small Integrin-Binding Ligand N-linked Glycoproteins (SIBLINGs), within recombinant amelogenin which resulted in guided HAP growth [16]. Hierarchical HAP growth on elastin-like recombinamer scaffolds shows the high level of precision and control that can be achieved with in vitro biomineralization [17,18]. However, guided uniaxial HAP growth along amelogenin scaffolds with a mineral promoting enamel protein, as occurs in vivo during mineralized tissue formation, has not been achieved with in vitro models. Herein, we describe a biomimetic approach involving two enamel-inspired recombinant proteins used in combination to guide uniaxial HAP formation. The interaction between amelotin, as a mineral promoter, and a previously described nanoengineered (recombinant) amelogenin (NA) protein, [5] as a scaffold, to guide mineral growth was examined under mineralizing conditions. A more detailed understanding of the molecular processes at play during protein-guided HAP formation may enable the development of mineralized tissue regeneration and treatment options.

## 2. Results and Discussion

Previously, recombinant human amelogenin (rH174) has been shown to self-assemble into nanoribbons in acidic solutions (pH 4.5–6.5) containing Ca^2+^ and phosphate ions [19]. Although rH174 nanoribbons are useful as scaffolds in guided HAP growth, the structure formation kinetics were highly variable, at times taking up to six months for nanoribbons to form. To address this variability, a nanoengineered amelogenin protein with an additional 12 amino acids [(KTKR)_3_] at its C-terminus was designed [5]. Incubation of NA in calcium phosphate (CaP) solutions at 37 °C resulted in nanoribbons and bundles of nanoribbons, detected within 3 days following initial assembly [5]. The dimensions of NA nanoribbons were comparable to those obtained with recombinant amelogenin (rH174) after 21 days of incubation, as characterized by optical microscopy (OM), transmission electron microscopy (TEM), scanning electron microscopy (SEM), and atomic force microscopy (AFM) images (Supplementary Figure S1). Despite the presence of Ca^2+^ and phosphate ions, under the conditions investigated, NA assemblies did not show any appreciable mineral formation.

**Figure 1:**
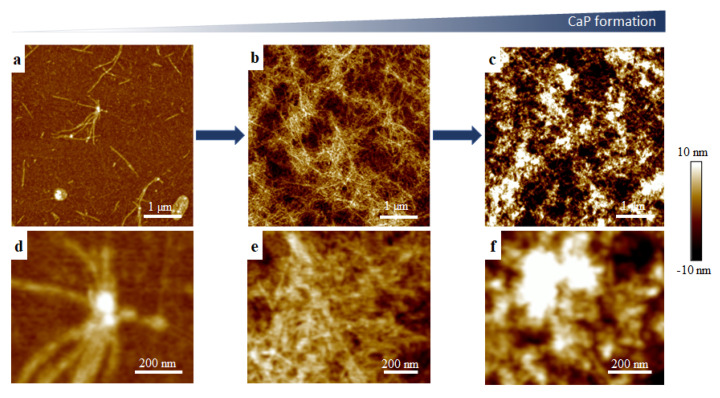
Tapping mode AFM in ambient conditions of amelotin self-assembly in calcium phosphate solutions after (**a**,**d**) 7, (**b**,**e**) 21 and (**c**,**f**) 28 days post initial assembly and incubation at 37 °C.

A biological role as a mineral promoter has been described for amelotin, however studies on amelotin self-assembly are scarce [20]. The self-assembly behavior of amelotin protein alone in CaP solutions was investigated prior to incubation experiments with NA scaffolds. In the present study, protein assembly and mineral formation were found to occur concurrently under the mineralizing conditions investigated. As shown in Figure 1 and Supplementary Figure S2, amelotin self-assembled to yield nanospheres that coalesced into fibers, and eventually became covered with mineral deposits over a period of 28 days of incubation, as characterized by AFM. Crystal precursors containing Ca^2+^ and phosphate ions have been previously suggested to be spherical when associated with an enamel matrix protein [21]. In the case of amelotin self-assembly, a gradual morphological transition was observed under conditions appropriate for CaP mineral formation. In contrast, when incubated in deionized water as a control, amelotin yielded only globular structures after 14 days of incubation (Supplementary Figure S3 and Table S1). Globular amelotin assemblies were also reported previously under neutral buffer conditions, which showcases the effect of pH and ionic strength on the morphology of amelogenin assemblies [22]. Though these data also show that amelotin is capable of mineral growth in vitro, mineral deposition was non-uniform. Therefore, we introduced a scaffold composed of self-assembled NA nanoribbons in an effort to promote guided HAP growth.

**Figure 2:**
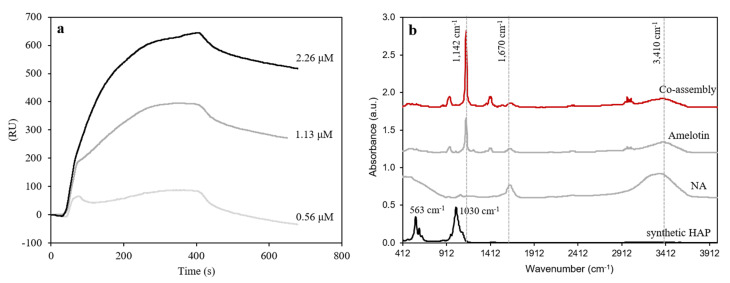
(**a**) SPR characterization of 2.26 μM amelotin on a NTA chip, with NA protein as analyte with a concentration of 2.26 (BMax 129.56°), 1.13 (BMax 415.89°) and, 0.56 μM (BMax 645.37°). The dissociation constant was calculated to be 1.25 × 10^−7^ ± 4.34 × 10^−9^, 1.61 × 10^−7^ ± 2.43 × 10^−9^ and 1.75 × 10^−7^ ± 8.29 × 10^−9^, respectively, as described in the Supplementary Materials. (**b**) FTIR examination of NA protein, amelotin, co-assembly and synthetic hydroxyapatite crystals. The main mineral observed in the co-assembly samples was hydroxyapatite.

Previously, amelotin and amelogenin (rH174) proteins did not show strong interactions with one another in a yeast two-hybrid analysis [23]. In light of the modified amelogenin sequence used in the present study, surface plasmon resonance (SPR) measurements were performed to test the binding affinity between the NA and amelotin. An average dissociation constant (K_D_) of 1.76 × 10^−7^ ± 5.02 × 10^−9^ measured during these experiments (Figure 2a) suggests a much stronger binding affinity than reported previously. It is likely that the additional +9 charge of NA compared to amelogenin (rH174) promotes stronger binding to amelotin through ionic interactions.

Fourier-transform infrared (FTIR) spectroscopy analyses showed structural changes in NA and amelotin upon self-assembly and HAP formation (Figure 2b). Hydroxyl groups were recorded as broad peaks at 3410 cm^−1^ in all protein samples but with a decreased intensity, presumably due to close association with HAP. Moreover, the peak corresponding to PO_4_^3−^ groups in bending mode [24] appeared at approximately 1140 cm^−1^. Here, the latter value is considerably shifted compared to the synthetic HAP sample. These results show that when self-assembled amelotin is incubated with NA nanoribbons, the two proteins interact in a manner that promotes HAP mineralization.

**Figure 3:**
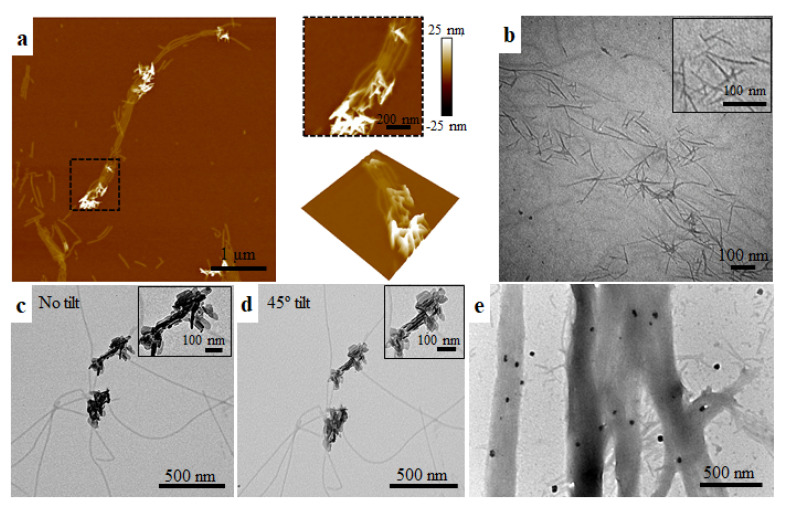
Co-assembly and mineralization of NA and amelotin at pH 5. (**a**) AFM and (**b**) TEM images of needle-like mineral growth along NA protein nanoribbons 20-min post co-incubation with amelotin. (**c**) Calcium phosphate mineral deposition organized along NA protein nanoribbons. (**d**) Figure 2c rotated 45° showing that the minerals are oriented along NA nanoribbons. (**e**) TEM of immunolabeled amelotin deposited along NA protein nanoribbons.

Further mineralization experiments were performed to evaluate the ability of amelotin to promote HAP formation in the presence of NA scaffolds for guided mineral growth. First, NA was assembled into nanoribbons in CaP solution at pH 5, then combined with amelotin at 1:1, 10:1 and 100:1 molar ratio. The turbidity of the solutions, indicative of the formation of mineral precipitates, was positively correlated with the amount of amelotin added (Supplementary Figure S4). At pH 6.5, there is a decrease in turbidity over time, likely due to mineral precipitation and deposition. The 100:1 ratio and 20-min incubation time were selected for further experiments in an effort to minimize spontaneous mineral precipitation. AFM and TEM images obtained during the characterization of amelotin-triggered mineralization on NA nanoribbons at pH 5 are shown in Figure 3. These results are generally consistent with guided HAP formation along NA nanoribbons, where thin and elongated HAP crystals were observed at the ends of nanoribbon agglomerates and along individual nanoribbons (Figure 3a–c). HAP crystals obtained with the co-incubated proteins had an average height of 15.1 ± 8.2 nm, which is more than 300% higher than that of individual nanoribbons (3.9 ± 1.0 nm) (Supplementary Figure S5). Moreover, the observed crystals had a compact and oriented shape localized on the nanoribbons, unlike the mineral formed with amelotin alone (Figure 1). The location of the HAP crystals along the top of the NA nanoribbons was confirmed by rotating the TEM samples by a 45° angle and corroborated by optical microscopy (Figure 3d,e and Supplementary Figure S6).

Immunogold labeling was performed to assess the distribution of amelotin during mineralization experiments involving both NA nanoribbons and amelotin. As shown in Figure 3e, the immunogold labeling results suggest that amelotin is present along the NA nanoribbons. Negative control samples lacking the primary antibody were used to confirm labeling protocol specificity (Supplementary Figure S7). It is worth noting that HAP crystals were not observed during imaging due to the high number of wash steps necessary during sample preparation for immunogold labeling. Collectively, these data suggest that an interaction between amelotin and pre-formed NA nanoribbons may promote guided HAP mineral growth.

**Figure 4:**
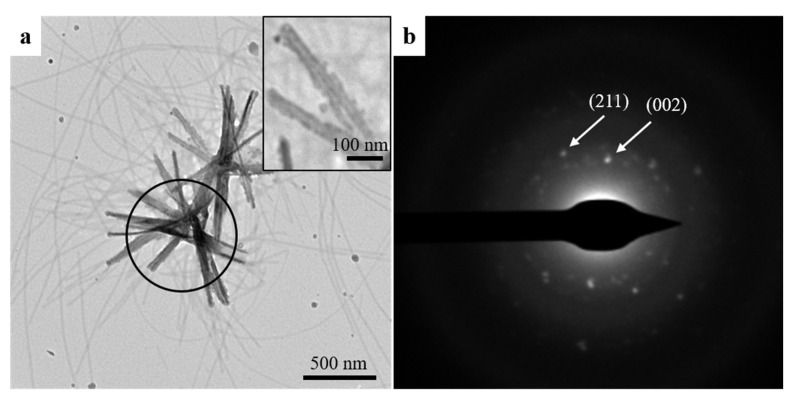
(**a**) Bright-field TEM image of hydroxyapatite organized along NA nanoribbons. (**b**) SAED pattern indicates characteristic crystallographic planes (211) and (002) for hydroxyapatite.

There was no evidence of mineralization in nanoribbon samples without added amelotin under the same conditions (Supplementary Figure S1), although HAP crystals have been obtained previously using amelogenin ribbons to guide mineral growth under more alkaline conditions [5]. The HAP crystals detected in this previous study were at a pH of 6.5 and were randomly deposited and at isolated locations, while extensive mineralization is reported here in the presence of amelotin. At pH 6.5, the combination of NA nanoribbons and amelotin also resulted in HAP growth (Figure 4a), however, the resulting crystal morphology was different. While elongated crystals with sharp edges were obtained at pH 5, a rod-like morphology with blunt edges was obtained at pH 6.5, resembling the structure of HAP rods found in enamel. Selected area electron diffraction (SAED) analysis confirmed the presence of the (002) and (211) characteristic crystallographic planes of HAP (Figure 4b). Therefore, these data agree with our hypothesis that NA nanoribbons can support and guide the growth of amelotin-promoted HAP. Incubation of amelotin under these conditions yielded thin fibrils bearing mineral deposits, observable without staining (Supplementary Figure S8), similar to the results obtained at pH 5.

As a control, we conducted similar experiments with NA nanoribbons and polyaspartic acid (pAsp), a known mineral promoter, instead of amelotin. pAsp is an amorphous mineral carrier that has been previously shown to promote in vitro HAP growth on recombinant amelogenin (rH146), DNA and demineralized dentin through the PILP process [25,26,27]. As observed by AFM and as previously described [28], pAsp assembles into nanospheres in CaP solutions (Supplementary Figure S9). In the absence of NA nanoribbons in solution, pAsp was randomly scattered across the surface (Supplementary Figure S9). When pAsp nanospheres were co-incubated with nanoribbons, they deposited along the length of individual nanoribbons, resembling pearls on a necklace (Supplementary Figure S9), and on top of NA nanoribbon aggregates (Supplementary Figure S10).

This is likely due to ionic interactions between the positively charged C-terminus of NA and negatively charged pAsp. Therefore, this result demonstrates that the scaffolding property of NA nanoribbons is not limited to amelotin and can be applied to other mineral carriers such as pAsp. However, the formed mineral did not have the high aspect ratio as observed with NA and amelotin assemblies. It is possible that the material formed is amorphous calcium phosphate (ACP), since it is typically a precursor of HAP in biomineralization processes [8,29]. Future studies will investigate the presence of amorphous precursor phases on NA nanoribbons HAP growth.

## 3. Materials and Methods

Protein preparation: N-terminally His_6_ tagged recombinant human amelotin (AMTN) and NA (also named rH174-(KTKR)_3_ and rH174(+9)) [5] proteins were synthesised and purified using previously published protocols [22,30].

SDS-PAGE electrophoresis: Protein samples were characterized using 12% SDS-PAGE in tris-glycine-SDS buffer at 180 V for 1 h at room temperature (Supplementary Figure S11).

Protein self-assembly: Briefly, 0.2 mg of amelotin or 2 mg NA protein were dissolved in 1 mL of a solution containing 34.1 mM CaCl_2_ and 20.9 mM NaH_2_PO_4_ at pH 5.0. The solution was placed in a partially opened vessel in a humidified incubator at 37 °C and concentrated by evaporation to approximately 20% of its original volume. The solution was then transferred to a closed container and placed in a humidified incubator at 37 °C for up to 4 weeks. Samples were aliquoted from this solution at 7, 14, 21, and 28 days after the initial assembly for further characterization [5,19]. All experiments were performed in triplicates.

Co-assembly of NA and amelotin: After self-assembly, amelotin solution was co-incubated with NA nanoribbons to achieve a molar ratio of 1:1, 10:1, or 100:1, as required, at 37 °C for 20 min.

Surface Plasmon Resonance (SPR): Experiments were performed using the OpenSPR instrument and NTA sensor chip (Nicoya Lifesciences, Kitchener, ON, Canada). Thus, 10 mM Tris-HCl (pH 7.4) was used as the running buffer, and all proteins were diluted in this running buffer. Experiments were performed at pH 7.4 in an effort to avoid protein aggregation, and to test individual protein-protein binding affinity. Hence, 200 µL of amelotin solution (2.26 µM) were injected into the sensor chip for immobilization on the Ni activated NTA chip. 0.56, 1.13 and 2.26 µM solutions of NA were injected as the analyte over the immobilized amelotin on the chip surface at the speed of 25 µL/min. Data acquired by SPR was analyzed using TraceDrawer software (Nicoya Lifesciences, version 1.8.1), assuming a 1:1 molar ratio for the interaction.

Optical Microscopy (OM): Protein morphology and mineralization were examined by drop casting 10 μL of NA nanoribbons and co-incubated NA-amelotin solutions on glass slides and imaged using a Zeiss Primo Vert optical microscope connected to an Axio Cam ERc 5s camera.

Atomic Force Microscopy (AFM): Samples were prepared by drop casting 5–10 μL of sample solution on mica (V-1 quality, Electron Microscopy Sciences, Hatfield, PA, USA) and incubating at room temperature for 30 min in a humidified chamber. The samples were then washed with 25 μL of deionized water and gently dried with compressed air. Imaging was carried out in tapping mode in ambient conditions on a MultiMode AFM with a Nanoscope III controller (Digital Instruments, Inc., Santa Barbara, CA, USA) using OTESPA-R3 cantilevers (Bruker, Billerica, MA, USA). Height measurements were calculated using the Nanoscope Analysis software (Bruker, version 2.0).

Scanning Electron Microscopy (SEM): Following AFM preparation, samples were placed on aluminum stubs and sputter-coated with a 2-nm Pt layer using a Fisons Polaron SC515 sputter coater. The external morphology of non-mineralized and mineralized proteins was determined using a FEI Inspect F-50 FE-SEM operating in high-vacuum mode at 5 kV.

Transmission Electron Microscopy (TEM): Samples were prepared by dropping 5 μL of sample solution on a glow discharged carbon-coated copper grid (Electron Microscopy Sciences, Hatfield, PA, USA) and incubated at room temperature for 60 s. Next, the grids were washed twice using an inverted deionized water drop. Excess liquid was removed by capillary action with a filter paper and the sample was air-dried prior to imaging. TEM images using the bright-field mode and selected area electron diffraction (SAED) patterns were acquired using a TEM (Hitachi H-7000, Tokyo, Japan) operating at 75 kV.

Immunogold labelling: Samples were fixed with 4% paraformaldehyde and 0.1% glutaraldehyde in phosphate buffer saline (PBS) at room temperature for 2 h. Fixed samples were then blocked with 4% BSA at room temperature for 1 h. Immunogold labelling probing for amelotin was performed on the grid by incubating samples with anti-mouse amelotin primary antibodies (1:100 dilution in PBS) overnight at 4 °C [12,22], followed by 15-nm-diameter gold conjugated to anti-rabbit secondary antibodies (Electron Microscopy Sciences, Hatfield, PA, USA) (1:100 dilution in PBS) incubated at room temperature for 1 h. Samples were washed 4× with PBS between each step. Grids were then imaged by TEM using a FEI Tecnai F20 microscope.

Fourier transformation infrared spectroscopy (FTIR): FTIR was used to determine NA and amelotin structure, and to characterize the mineral formed during protein co-assembly at pH 5. Hydroxyapatite nanocrystals (Berkeley Advanced Biomaterials, Inc., Berkley, CA, USA) were used as a control. Spectra were collected with a Thermo Scientific FTIR Nicolet iS20 instrument having a germanium crystal and a resolution of 2 cm^−1^. Each spectrum was collected after 32 scans and analyzed using the OMNIC software.

## 4. Conclusions

In summary, we demonstrated for the first time that two recombinant proteins, amelotin and NA, can promote guided HAP mineralization along self-assembled nanostructures in vitro. In this study, we described the self-assembly of amelotin into supramolecular structures in the presence of Ca^2+^ and phosphate ions, and we showed that amelotin maintains its mineral promoting properties upon interaction with NA nanoribbon scaffold. These results provide insights concerning mechanisms at play during guided HAP formation on protein scaffolds in vitro. Parallel mechanisms may occur during mineralized tissue formation, in particular during enamel maturation. However, the detailed mechanisms guiding the formation of the highly complex structure of enamel are still under investigation. Further studies on the mechanisms at play during guided HAP growth are crucial for the development of artificial biomaterials capable of mineralized tissue regeneration.

## Data Availability

Data are available upon request.

## Supplementary Materials

The following are available online at https://www.mdpi.com/article/10.3390/ijms222212343/s1.
