# Supplementary material for: Uniaxial Hydroxyapatite Growth on a Self-Assembled Protein Scaffold
Source: Int J Mol Sci. 2021 Nov 15;22(22):12343. doi: 10.3390/ijms222212343 (PMC8620880; doi:10.3390/ijms222212343)
Supplement: Supplementary file 1 [file ijms-22-12343-s001.zip › ijms-1458521-supplementary.pdf]

Uniaxial Hydroxyapatite Growth on a Self-Assembled Protein Scaffold

SUPPLEMENTARY INFORMATION

Alexander L. Danesi [1,†], Dimitra Athanasiadou [1,†], Ahmad Mansouri [1], Alina Phen [1], Mehrnoosh Neshatian [1], James Holcroft [1], Johan Bonde [2], Bernhard Ganss [1,3] and Karina M. M. Carneiro [1,3,*]

[1] Faculty of Dentistry, University of Toronto, Toronto, ON M5G 1G6, Canada; a.danesi@utoronto.ca (A.L.D.); dimitra.athanasiadou@utoronto.ca (D.A.); ahmad.mansouri@mail.utoronto.ca (A.M.); alina.phen@mail.utoronto.ca (A.P.); mehrnoosh.neshatina@mail.utoronto.ca (M.N.); james.holcroft@utoronto.ca (J.H.); b.ganss@dentistry.utoronto.ca (B.G.)
[2] Division of Pure and Applied Biochemistry, Center of Applied Life Sciences, Lund University, 223 62 Lund, Sweden; johan.svensson_bonde@tbiokem.lth.se
[3] Institute of Biomedical Engineering, University of Toronto, Toronto, ON M5S 3G9, Canada
* Correspondence: karina.carneiro@utoronto.ca
† Authors with equal contribution.

1. Experimental Methods

Protein preparation. Six-times (6x) His tagged recombinant human amelotin (AMTN) was synthesised using a previously published protocol[1]. In this method, the recombinant amelotin was expressed in *Escherichia coli* (pET-15b expression system, Novagen, Merck KGaA, Darmstadt, Germany) and affinity purified on an Ni-NTA agarose column (Qiagen, Valencia, CA, USA) to near homogeneity[1]. 0.2 mg of amelotin was dissolved in 1 mL solution containing 34.1 mM $CaCl_2$ and 20.9 mM NaH_2PO_4 at pH 5.0 or 6.5, as required. The solution was placed in a partially opened vessel in a humidified incubator at 37°C, and concentrated by evaporation ~5x. The solution was then transferred to a closed container and placed in a humidified incubator at 37°C for up to 4 weeks. Samples were aliquoted from this solution at 7, 14, 21, and 28 days for further characterization. The experiment was performed in triplicates.

Human recombinant amelogenin with a C-terminus modification [rH174-(KTKR)$_3$, also known as rH174(+9)] and nanoengineered amelogenin (NA) protein, was expressed in *E. coli* and purified using a one-step acid precipitation treatment[2], followed by dialysis against 0.05% HAc and lyophilization. For self-assembly, a previously described protocol was used[3]. Briefly, 2 mg of amelogenin was dissolved in 1 mL solution containing 34.1 mM $CaCl_2$ and 20.9 mM NaH_2PO_4 at pH 5.0 or 6.5, as required. The solution was placed in a partially opened vessel in a humidified incubator at 37°C, and concentrated by evaporation ~5x. The solution was then transferred to a closed container and placed in a humidified incubator at 37°C for up to 4 weeks. Samples were aliquoted from this solution for further characterization. The experiment was performed in triplicates. We focused on pH ranging from 4 to 6.5 because previous reports have shown amelogenin ribbons to form within this range[4]. Samples were aliquoted from this solution for characterization after 7, 14, 21, and 28 days. All experiments were performed in triplicates.

SDS-PAGE electrophoresis. Protein samples were characterized using 12% SDS-PAGE in tris-glycine-SDS buffer at 180 V for 1 hour at room temperature (BioRad Protean II system). Reagents were purchased from Sigma Aldrich and the gel was run according to the manufacturer's protocol. A Novex benchmark protein ladder was used (Cat. No. 10747-012). Biorad Oriole Fluorescent stain was used to visualize the proteins (Supplementary Fig. S11).

pAsp self-assembly: 13.5 mg of pAsp were dissolved in 10 mL of deionized water. Solution was then sonicated for 12 minutes (10 minutes before each use).

Co-assembly of NA protein and pAsp: 1 μL of assembled pAsp and 9 μL of self-assembled NA protein were incubated at room temperature for 20, 40, or 60 minutes. The co-assembly was then characterized by AFM and TEM.

Co-assembly of NA protein and amelotin: After 2-3 weeks post-assembly, NA and amelotin protein solutions were added together to achieve a molar ratio of 10:1 or 1:1, and incubated at 37ºC for 20 minutes.

Nanoengineered amelogenin (NA) protein sequence:[3]

NH_2 – PLPPHPGHPGYINFSYEVLTPLKWYQSIRPPYPSYGYEPMGGWLHHQIIPVLS
QQHPPTHTLQPHHHIPVVPAQQPVIPQQPMMPVPGQHSMTPIQHHQPNLPPPAQQPYQP
QPVQPQPHQPMQPQPPVHPMQPLPPQPPLPPMFPMQPLPPMLPDLTLEAWPSTDKTKRE
EVDKTKRKTKRKTKR – COOH

Amelotin (AMTN) sequence:[1]

NH_2 – HHHHHHSHMLPQLKPALGLPPTKLAPDQGTLPNQQQSNQVFPSLSLIPLTQMLTL
GPDLHLLNPAAGMTPGTQTHPLTLGGLNVQQQLHPHVLPIFVTQLGAQGTILAAEELPQI
FTSLIIHSLFPGGILPTSQAGANPDVQDGSLPAGGAGVNPATQGRLPTPSGTDDDFAVTTP
AGIQRSTHAIEEATTESANGIQ – COOH

SPR, K_D calculations:

Data was generated with OpenSPR and was analyzed using TraceDrawer software. Interval of interest was defined according to the software manual. The start point was set to the time point when the first concentration change has been captured and the end timepoint was set to the end of association time. One To One Diffusion Corrected model was used from the library of the software to fit a curve to the signal. The small χ^2 value indicated that the fitted curve closely approximated the signal.

Tables and Figures:

Supplementary Table S1. Self-assembled amelotin fibril AFM measurements.

	7 days post-assembly (nm)	14 days post-assembly (nm)
**Width**	13.1 ± 5.2	40.0 ± 17.0
**Height**	1.2 ± 0.7	3.2 ± 1.8

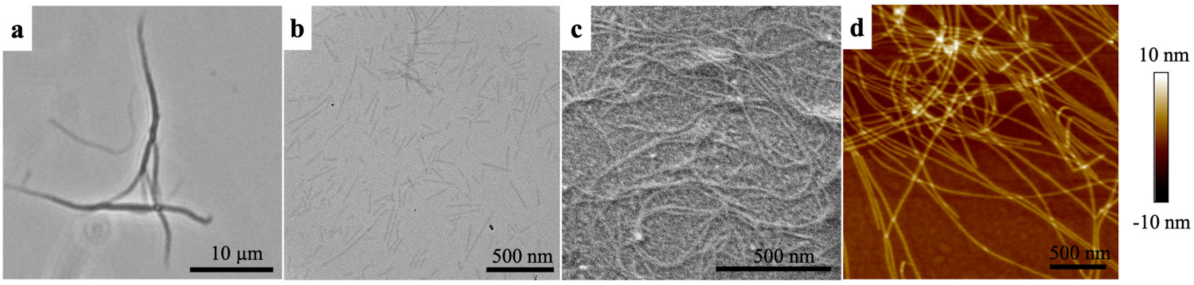

Figure S1. Optical microscopy, TEM, SEM and AFM characterization of self-assembled NA nanoribbon bundles over 21 days post initial assembly at pH 5.

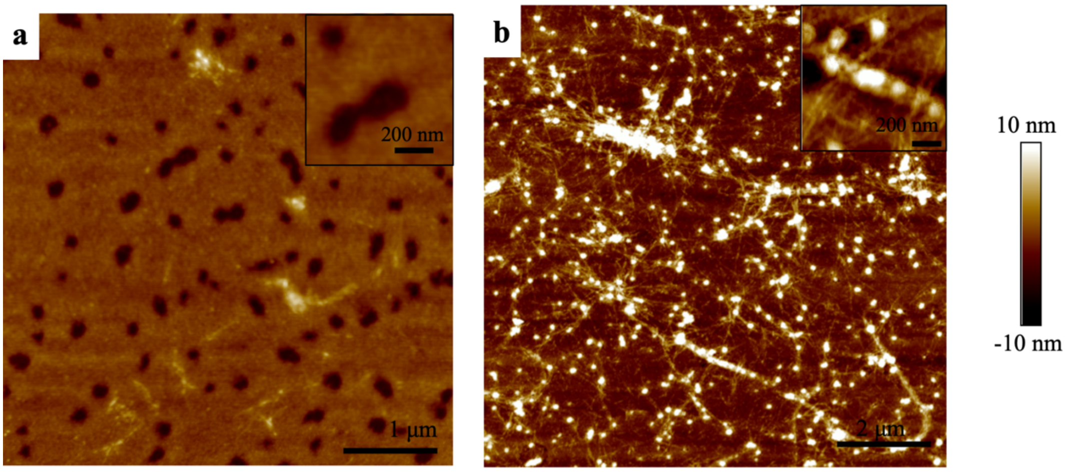

Figure S2. AFM images of self-assembled amelotin (pH 5) showing nanosphere formations at day 4 and mineral growth after 14 days post initial assembly and incubation at 37ºC.

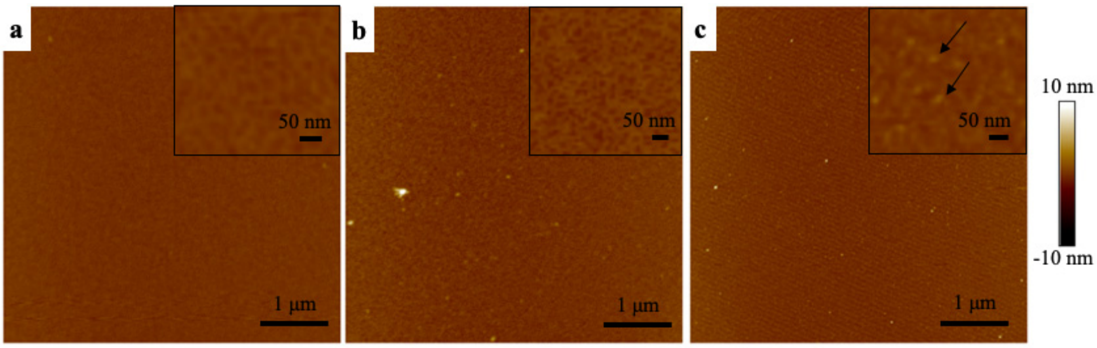

Figure S3. Self-assembly of amelotin in deionized water at pH 5 imaged by AFM: (a) 4 days, (b) 7 days, and (c) 14 days post initial assembly and incubation at 37ºC. Arrows indicate globular structures.

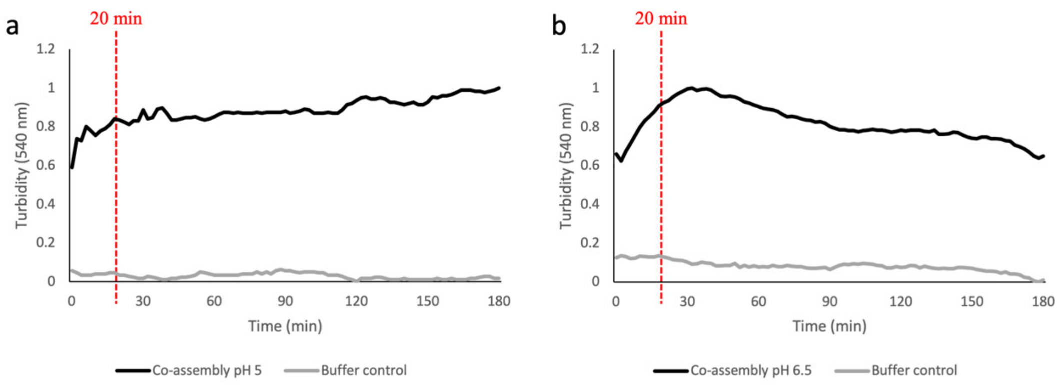

Figure S4. Turbidity (absorbance measured at 540 nm, 37ºC) of solutions containing NA protein and amelotin protein co-assembly (black) and buffer only (grey) at (a) pH 5 and (b) 6.5.

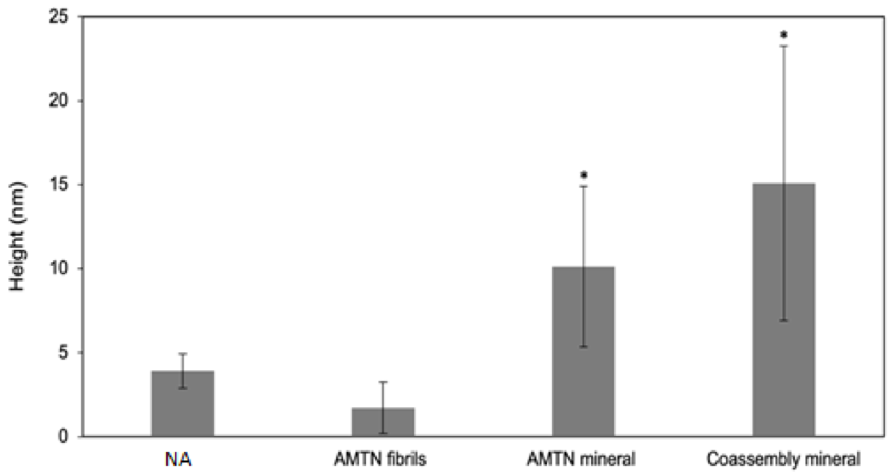

Figure S5. Height measurements of self-assembled proteins (NA and amelotin) and mineral produced based on AFM images. A t-test is used here and data are presented as mean ± standard deviation ($n \geq 50$). *$p < 0.05$ (two-tailed) for amelotin mineral and co-assembly mineral.

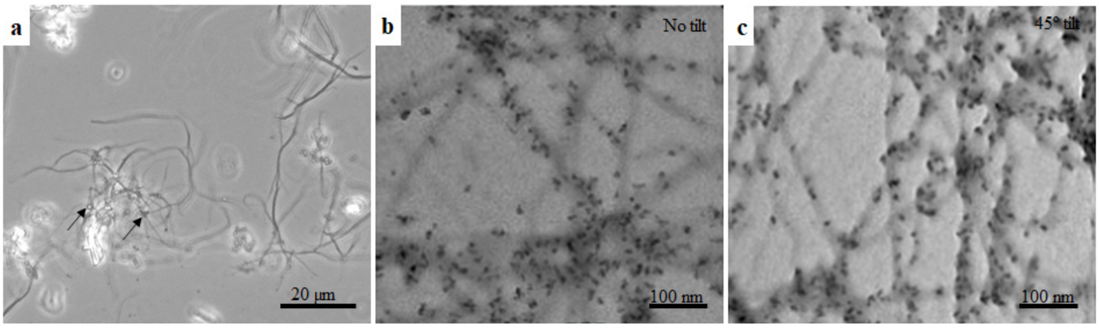

Figure S6. Optical microscopy (a) and TEM (b and c) of hydroxyapatite crystals (arrows) grown along NA assemblies.

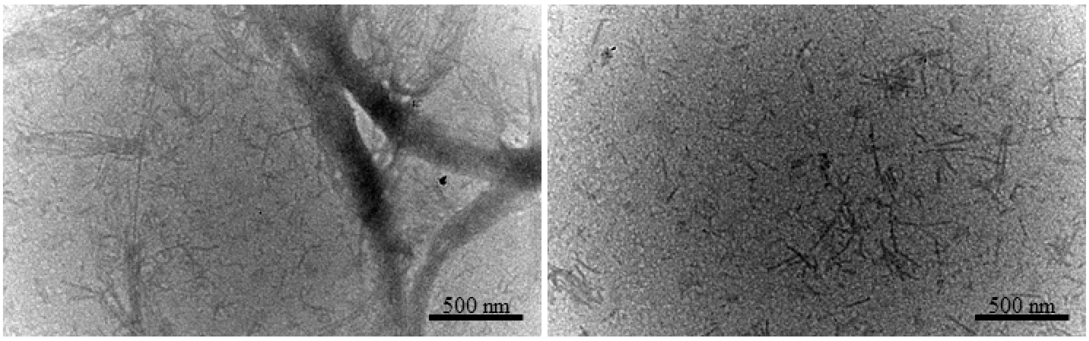

Figure S7. TEM images of immunogold labeling of control sample containing NA nanoribbons without amelotin.

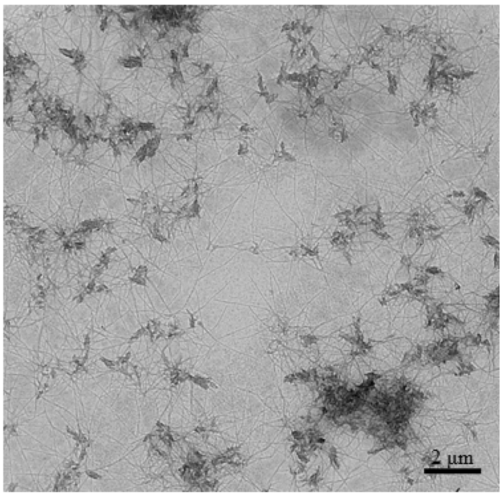

Figure S8. TEM image of self-assembled amelotin (pH 6.5) showing mineral growth after 14 days post initial assembly.

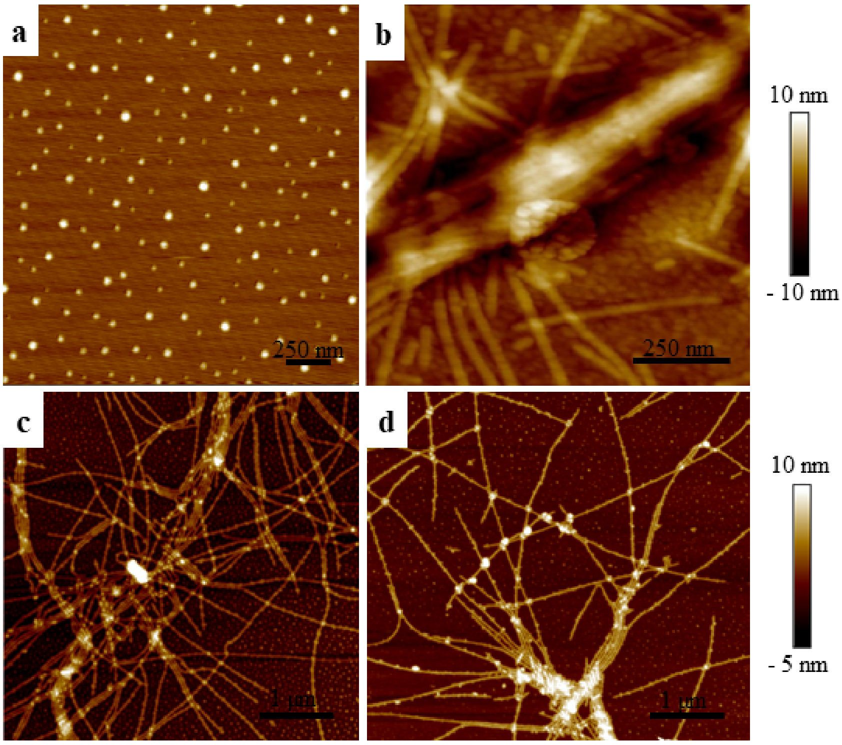

Figure S9. AFM images of (a) pAsp, (b) co-assembly of pAsp and NA protein, and mineralization after (c) 20 min and (d) 1 hour.

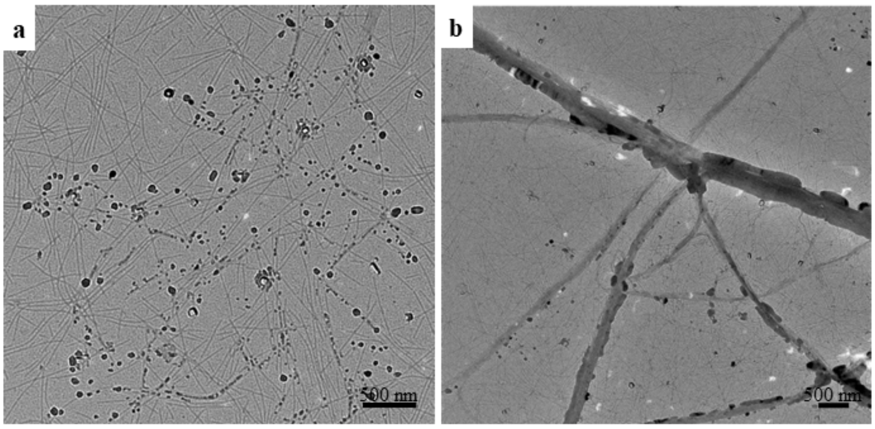

Figure S10. TEM images of 20-minute co-assembly between pAsp and NA protein: (a) pAsp droplets deposited along the length of individual nanoribbons, and (b) dense mineral deposition observed along bundled nanoribbons.

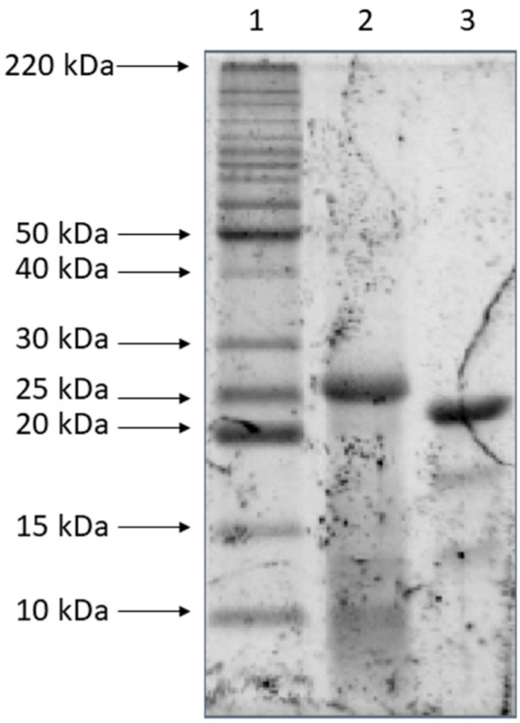

Figure S11. 12% SDS PAGE gel with: Lane 1 - Novex Benchmark Protein ladder; Lane 2 - NA protein; Lane 3 - amelotin protein.

References:

1. Abbarin, N., San Miguel, S., Holcroft, J., Iwasaki, K. & Ganss, B. The enamel protein amelotin is a promoter of hydroxyapatite mineralization. *J. Bone Miner. Res.* **30**, 775-785, doi:10.1002/jbmr.2411 (2015).

2. Svensson Bonde, J. & Bulow, L. One-Step Purification of Recombinant Human Amelogenin and Use of Amelogenin as a Fusion Partner. *PLoS One* **7**, e33269, doi:10.1371/journal.pone.0033269 (2012).

3. Carneiro, K. M. M. *et al.* Amyloid-like ribbons of amelogenins in enamel mineralization. *Sci. Rep.* **6**, 23105, doi:10.1038/srep23105 (2016).

4. Martinez-Avila, O. *et al.* Self-Assembly of Filamentous Amelogenin Requires Calcium and Phosphate: From Dimers via Nanoribbons to Fibrils. *Biomacromol.* **13**, 3494-3502, doi:10.1021/bm300942c (2012).
